# Impact of a new medical network system on the efficiency of treatment for eating disorders in Japan: a retrospective observational study

**DOI:** 10.1186/s13030-017-0113-9

**Published:** 2017-10-01

**Authors:** Junko Moriya, Mami Kayano, Kazuhiro Yoshiuchi

**Affiliations:** Department of Stress Sciences and Psychosomatic Medicine, Graduate School of Medicine, The University of Tokyo, 7-3-1 Hongo, Bunkyo-ku, Tokyo, 113-8655 Japan

**Keywords:** Eating disorders, Outpatient treatment, Care pathways, Medical community network

## Abstract

**Background:**

In Japan, patients generally have free access to any hospital or clinic. This could lead to reduced efficiency in the treatment for eating disorders (EDs) because there are only a limited number of doctors who can treat ED patients. The objectives of this study were to examine the efficiency of a new trial system for consultation and appointments, a medical community network (MCN), in outpatient treatment for EDs. MCN schedules appointments for the first visit only by referral from another medical institution, not by patients themselves.

**Methods:**

We analyzed the data of 342 outpatients (mean age = 28.9 ± 9.9 years; 328 female and 14 male) who visited the ED clinic at the University of Tokyo Hospital for the first time between January 2009 and July 2012 to investigate possible differences in treatment efficacy between the new (MCN+) system and the conventional (MCN-) system, which accepts reservations directly from patients.

**Results:**

The no-show rate for MCN+ patients (0.8%) was significantly lower than that for the MCN- group (17.8%) (*p* < 0.001). MCN+ patients had a significantly shorter waiting period (8.4 days) for the first visit compared to MCN- patients (35.5 days, p < 0.001). In addition, the MCN+ group had a much higher rate of successive visits to the clinic (*p* < 0.05).

**Conclusion:**

This new consultation system using a medical community network provided more efficient treatment for ED than did the appointment system in which the patients made their appointments by themselves.

## Background

The number of patients with eating disorders (EDs) in Japan has increased ten-fold since 1980 and is still increasing each year [1]. According to a study in 2007 [2], the Japanese ED prevalence rates per 100,000 people were 12.1 for anorexia nervosa (AN) and 18.2 for bulimia nervosa (BN). The clinical presentation of EDs in Japan appears to be similar to that of Western cultures [3]. However, the Japanese clinical system has two major problems. First, Japan has only a few hospitals that have ED specialized clinics, and the number of physicians who can treat patient with ED is quite limited [4]. Second, unlike Europe and the United States where psychiatry and psychotherapy are the centers of treatment, ED patients in Japan visit various departments, such as psychiatry, psychosomatic medicine, internal medicine, pediatrics, obstetrics and gynecology, emergency medicine, and dentistry [5]. As a result, ED patients in Japan have difficulty in obtaining proper and efficient treatment by specialists.

The medical system in Japan has aspects distinct from those of other countries [6]. In Japan, under “universal coverage” all medical costs, including prescribed drugs, are strictly controlled by the government, so that all patients receive high-quality medical services at a relatively low cost. Additionally, patients can visit any medical institution of their choice without referrals, including university hospitals. However, a major disadvantage of this system is its inefficiency, such as doctor-shopping and a mismatch between the need for and the supply of health-care resources. For example, even for low-risk operations, increasingly more patients now seek care from specialists in tertiary hospitals [7]. Due to the large number of patients visiting general hospitals for relatively minor problems, a shortage of medical resources is one of the most important issues in Japan. The large number of appointments per physician results in a long waiting time, which has become a serious problem [8]. An effective referral system would be beneficial to Japan and many other countries, as it can improve patient access to ED-specific treatment and care.

A previous study in the U.K. [9] showed that specialist outpatient services led to a significantly lower rate of admission for inpatient treatments and considerably higher consistency of care. The study suggested that the presence of experts in outpatient service has a strong positive effect on diagnosis and the precise treatment of EDs. However, the system in the U.K. is much different than that of Japan because patients in Japan can visit any hospital without a referral by their family doctor. Thus, the findings of the previous study may not be directly applicable to Japan. Little is known about the impact of alternative systems in the treatment efficiency of EDs in Japan, although there have been several studies in Japan reporting various outcomes in the treatment of EDs [10–15].

In April 2005, we began using a medical community network (MCN) consultation system to enhance partnerships with other medical institutions and to improve the matching of patients to our tertiary hospital. Therefore, the objective of the present study was to examine the efficacy for EDs of this new MCN consultation and appointment service at a university hospital in Japan.

## Methods

### Two options for scheduling an appointment for a first visit to an ED clinic

The Department of Psychosomatic Medicine at the University of Tokyo Hospital has an outpatient ED clinic and an inpatient unit. The clinic requires patients to schedule an appointment for their first visit, which can be done in one of two ways: via MCN and only by referral from their primary physician (MCN+) or via the usual reservation system by themselves (MCN-). Appointments via MCN- included both patients with and without a referral by their former doctor and patients who made a reservation directly by phone call. The appointment via MCN+ required the former doctor to fax a reservation application form to the MCN department, and the doctor was sent a confirmation reply by fax.

### Study participants

We analyzed the data of 342 outpatients (328 female, 14 male) who visited our ED clinic for the first time between January 2009 and July 2012. As shown on Tables 1, 128 patients (124 female, 4 male) were assigned to MCN+ and 214 patients (204 female, 10 male) to MCN-.

**Table 1:** Participant characteristics

	ALL	MCN +	MCN -	MCN + vs. MCN -
Number patients with a reserved appointment(F/M)	342 (328/14)	128 (124/4)	214 (204/10)	
Number of patients with a first follow-up visit (F/M)	303 (290/13)	127 (123/4)	176 (167/9)	
No-show rate (F/M) (%)	11.1 (11.6/7.1)	0.8 (0.9/0)	17.8 (18.1/10.0)	p < 0.001
Waiting period from reservation to first visit (days)	24.5 ± 17.7	8.4 ± 5.7	35.5 ± 14.2	*p* < 0.001
Age (years)	28.9 ± 9.9	30.5 ± 11.1	27.9 ± 9.0	*p* = 0.073
Diagnosis				
AN-R	87 (28.8%)	46 (36.2%)	41 (23.3%)	p < 0.05
AN-BP	80 (18.8%)	33 (26.0%)	47 (26.7%)	*p* = 0.92
BN-P	57 (18.8%)	18 (14.2%)	39 (22.2%)	p = 0.07
BN-NP	14 (4.6%)	4 (3.1%)	10 (5.7%)	*p* = 0.31
ED-NOS	34 (11.2%)	5 (3.9%)	29 (16.5%)	*p* < 0.01
Others	31 (10.2%)	21 (16.5%)	10 (5.7%)	p < 0.05
Treatment				
Only once	151 (49.8%)	49 (38.6%)	102 (58.0%)	p < 0.001
Outpatient treatment only	83 (27.4%)	46 (36.2%)	37 (21.0%)	p < 0.01
Inpatient treatment only				
Outpatient + inpatient	16 (5.3%)	11 (8.7%)	5 (2.8%)	*p* = 0.423
treatment	53 (17.5%)	21 (16.5%)	32 (18.2%)	*p* = 0.133

*MCN* medical community network, *F* female, *M* male, *AN-R* anorexia nervosa restricting type, *AN-BP* anorexia nervosa binge eating/purging type, *BN-P* bulimia nervosa pursing type, *BN-NP* bulimia nervosa non-purging type, *ED-NOS* eating disorders not otherwise specified

### Study protocol

On August 31st, 2012, we examined the following items stored in electronic medical records: “whether the patients actually kept their appointment”, “the duration from scheduling an appointment to the first visit”, “diagnosis based on the Diagnostic and Statistical Manual of mental Disorders (DSM-IV) [16]”, “the number of outpatient treatment sessions”, “the number of hospitalizations”, and “the present consultation situation”. The observation period was not different between the two groups. This study was approved by the Ethics Committee of the Graduate School of Medicine, The University of Tokyo.

### Statistical analysis

All analyses were performed using SPSS for Windows, ver. 20.0 J (IBM, Tokyo, Japan). Continuous data were analyzed by t-test. Categorical data were analyzed using the chi-square test. When there was a statistically significant difference among groups using the chi-square test, the data were further analyzed using residual analysis [17]. The criterion used for statistical significance was *p* < 0.05 in a two-tailed test.

## Results

### First visit

The no-show rate for the MCN+ patients (0.8%) was significantly lower than that of the MCN- patients (17.8%) (*p* < 0.001) (Table 1). In addition, the MCN+ group had a significantly shorter waiting period from the time of reservation to the first visit than the MCN- group (MCN+ vs. MCN-; 8.4 ± 5.7 days vs. 35.5 ± 14.2 days, p < 0.001).

### Diagnosis

Table 1 shows the diagnosis of the patients according to the criteria of DSM-IV. The 128 first-visit outpatients in MCN+ included 46 (36.2%) with the AN restricting type (AN-R), 33 (26.0%) AN binge eating/purging type (AN-BP),18(14.2%) BN purging type (BN-P), 4 (3.1%) BN non-purging type (BN-NP), 5 (3.9%) ED not otherwise specified (ED-NOS), and 21(16.5%) others. The diagnoses of the 214 first-visit outpatients in MCN- included 41 (23.3%) with the AN restricting type (AN-R), 47 (26.7%) AN binge eating/purging type (AN-BP), 39 (22.2%) BN purging type (BN-P), 10 (5.7%) BN non-purging type (BN-NP), 29 (16.5%) ED not otherwise specified (ED-NOS), and 10 (5.7%) others. The rate of patients with AN-R was significantly higher in MCN+ than in MCN- (*p* < 0.05).

### Treatment

Table 1 shows the number and type of treatment sessions provided: For MCN+ “only once” 49 (38.6%), “outpatient treatment only” 46 (36.2%), “inpatient treatment only” 11 (8.7%), and “outpatient and inpatient treatment” 21 (16.5%). For MCN- “only once” 102 (59.0%), “outpatient treatment only” 34 (19.7%), “inpatient treatment only” 5 (2.9%), and “outpatient and inpatient treatment” 32 (18.5%). In MCN-, 59% of the patients visited the ED clinics only once, which was significantly higher than the rate of 38.6% for MCN+ (*p* < 0.05).

### Treatment disposition

**Fig. 1:**
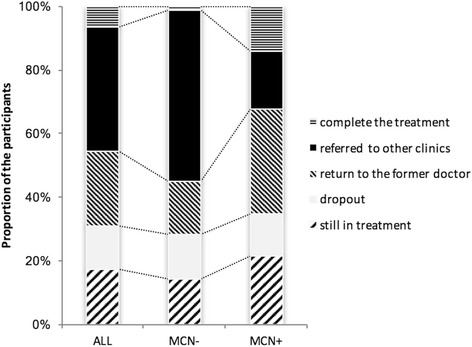
Treatment disposition. More MCN- than MCN+ patients were introduced to other clinics, mainly to the department of psychiatry (MCN- vs. MCN+; 54.0% vs. 18.1%, *p* < 0.05). More MCN+ patients completed treatment or returned to their former doctor (p < 0.05). MCN: medical community network

Figure 1 shows the treatment disposition of the participants. More MCN- than MCN+ patients were referred to other clinics, mainly to the department of the psychiatry (*p* < 0.05). By contrast, more MCN+ than MCN- patients completed the treatment or returned to their former doctor (*p* < 0.05).

## Discussion

The present study was a retrospective observational study on consultation and appointment pathways for ED treatment. The main contribution of the present study is to show significant differences in the no-show rate and in treatment disposition between the MCN+ and MCN- groups.

In MCN+, the waiting period before the first visit was significantly shorter and the appearance rate was significantly higher than for MCN-. Although the background of the patients who did not come to the first visit is unknown, one possible reason for the difference in waiting period was that appointment via MCN- was more easily done than by MCN+. MCN+ and MCN- were similar with regard to the number of reservations that could be accepted (two patients per week in MCN+ and two to four patients per week in MCN-). MCN- accepted appointments by patients with or without referrals by their former doctors. In addition, patients could make a reservation easily just by a phone call. On the other hand, the appointment via MCN+ required more effort by the patient’s former doctor. The shorter waiting period might have led to the high appearance rate in MCN+.

However, neither study focused on the waiting time between diagnosis and initial scheduling. A Japanese survey of the outcome of 477 ED patients at six institutions from 4 to 11 years after the first visit reported a recovery rate of 53%, partial recovery by16%, no change in ED status for 24%, and death in 7% of their cases [11]. A study of the outcome of 67 AN patients who received cognitive behavioral therapy reported a recovery rate of 57.1%, partial recovery by 14.3%, no change in ED for 24%, and worse outcomes for 14% of their patients [10]. The lower rate of kept appointments for the MCN- group could be a combination of the longer wait time and their increased severity of pathology. On the other hand, in the MCN+ group, the physician-referral system could be more efficient at getting people quickly into outpatient treatment. This is advantageous for patients over the long-term given that early intervention is generally associated with better outcome.

The MCN+ group had a much higher rate of successive visits after the first visit to the clinic. Satisfactory treatment compliance is also associated with better outcome. The present study showed that MCN+ had less mismatch between the need for and the supply of patient services. Although we did not inform our acceptance conditions to the primary care doctors or patients, we posted them on our website: "If violent self-harmful behavior persists or hallucinations/delusions are recognized, we will introduce the patient to the psychiatry department.” The primary care doctors who used the MCN might be strongly motivated, which may have led them to check the website. Thus, these primary care doctors were more informed about “the service that can be provided at the department of psychosomatic medicine”, which might have led to more efficient treatment service. On the other hand, for MCN- there were significantly more mismatching cases introduced to the psychiatry department because their main problem was a psychiatric disease other than ED. The most frequent cases were borderline personality disorder, bipolar disorder with poor impulse control such as self-injury, or schizophrenia with hallucinations and delusions. These results suggest that matching the need for and supply of health-care resource can be critical in providing effective and efficient treatment.

The proportion of AN-R was higher in the MCN+ group than MCN-. Many AN-BP patients undergo transition from AN-R [18]. Therefore, the doctors using MCN+ might have had strong motivation to treat eating disorder patients, which may have led to them finding patients at an earlier stage. However, future studies on this issue will be necessary.

There are several limitations to our study. First, we did not survey the reason the patients who had appointments did not appear at the clinic. The lower rate of appearance for the MCN- group could be a combination of the longer waiting period and their increased severity of pathology, or they might have sought care elsewhere. Second, we did not match the two groups regarding the severity of illness. In the present study, onset, duration of illness, and body mass index were not investigated. Therefore, the difference in pathology and severity between the two groups was not clear. Third, the doctors in charge of the two groups had almost the same years of experience (about four years) treating eating disorder patients and had received the same training program. However, the quality and intensity of treatment might not have been exactly the same between the two groups, although some of the doctors were involved with both MCN+ and MCN-. Fourth, we did not get treatment outcome data. Lastly, the new system is being used in all departments of The University of Tokyo Hospital. Future prospective longitudinal studies will be necessary to determine if the new system is efficient for all departments or only for ED.

## Conclusions

We have provided evidence that a new consultation and appointment system may contribute to the efficiency of consultation. Appointment scheduling by doctors may improve the treatment engagement in ED compared to appointments made by patients themselves.

## Abbreviations

AN: Anorexia nervosa
AN-BP: Anorexia nervosa binge eating/purging type
AN-R: Anorexia nervosa restricting type
BN: Bulimia nervosa
BN-NP: Bulimia nervosa non-purging type
BN-P: Bulimia nervosa purging type
ED: Eating disorder
ED-NOS: Eating disorders not otherwise specified
MCN: Medical community network
